# Modeling the phenotype of spinal muscular atrophy by the direct conversion of human fibroblasts to motor neurons

**DOI:** 10.18632/oncotarget.14641

**Published:** 2017-01-13

**Authors:** Qi-Jie Zhang, Jin-Jing Li, Xiang Lin, Ying-Qian Lu, Xin-Xin Guo, En-Lin Dong, Miao Zhao, Jin He, Ning Wang, Wan-Jin Chen

**Affiliations:** ^1^ Department of Neurology and Institute of Neurology, First Affiliated Hospital, Fujian Medical University, Fuzhou, China; ^2^ Fujian Key Laboratory of Molecular Neurology, Fuzhou, China

**Keywords:** direct reprogramming, fibroblast, induced motor neuron, spinal muscular atrophy

## Abstract

Spinal muscular atrophy (SMA) is a lethal autosomal recessive neurological disease characterized by selective degeneration of motor neurons in the spinal cord. In recent years, the development of cellular reprogramming technology has provided an alternative and effective method for obtaining patient-specific neurons in vitro. In the present study, we applied this technology to the field of SMA to acquire patient-specific induced motor neurons that were directly converted from fibroblasts via the forced expression of 8 defined transcription factors. The infected fibroblasts began to grow in a dipolar manner, and the nuclei gradually enlarged. Typical Tuj1-positive neurons were generated at day 23. After day 35, induced neurons with multiple neurites were observed, and these neurons also expressed the hallmarks of Tuj1, HB9, ISL1 and CHAT. The conversion efficiencies were approximately 5.8% and 5.5% in the SMA and control groups, respectively. Additionally, the SMA-induced neurons exhibited a significantly reduced neurite outgrowth rate compared with the control neurons. After day 60, the SMA-induced neurons also exhibited a liability of neuronal degeneration and remarkable fracturing of the neurites was observed. By directly reprogramming fibroblasts, we established a feeder-free conversion system to acquire SMA patient-specific induced motor neurons that partially modeled the phenotype of SMA in vitro.

## INTRODUCTION

Spinal muscular atrophy (SMA) is one of the most common autosomal recessive disorders in humans and has a high frequency of 1 of every 6,000 to 10,000 live births. The majority of SMA cases are attributable to the homozygous loss of the survival motor neuron gene (SMN1) in 5q13, which leads to the selective degeneration of motor neurons in the spinal cord and progressive muscular weakness and atrophy [1]. Based on the age at onset and the highest level of motor function, childhood onset SMA can be further divided into three types (SMA type I-III). Type I is the most severe, and these patients often develop muscle weakness before 6 months, never sit and die within the first 2 years due to respiratory failure. Due to the lack of currently available effective treatment, SMA continues to account for a large percentage of the genetically caused deaths in infancy.

Because SMA is a lethal genetic disease, the acquisition of patient-specific and SMA-related motor neurons is important. Recently, with the development of cell reprogramming technology, induced pluripotent stem cells (iPSs), which are reprogrammed from adult somatic cells via the overexpression of defined transcription factors, open a new and effective avenue for the generation of induced motor neurons in vitro [2–3]. Increasing amounts of research have employed iPS-derived neurons for SMA studies related to pathogenesis research and drug screening [4–5]. Similar to iPS technology, the direct conversion of non-neural cells (for example, fibroblasts) to neurons can be achieved by the forced expression of some lineage-specific factors. In 2010, Vierbuchen T and colleagues successfully converted mouse fibroblasts into neurons with three transcription factors, i.e., Ascl1, Brn2, and Myt1l [6]. Subsequently, these authors also converted human fibroblasts into functional neurons with ASCL1, BRN2, MYT1L, and NEUROD1 [7]. Subsequently, other groups also reported the direct conversion of fibroblasts into neural stem/progenitor cells that are self-renewing and capable of producing specific types of neurons, such as dopaminergic neurons, astrocytes, and oligodendrocytes [8–10]. Additionally, Son EY and colleagues directly converted mouse and human fibroblasts into motor neurons with 8 transcription factors, includingASCL1, ISL1, NEUROD1, BRN2, HB9, LHX3, MYT1L and NGN2, without passing through a proliferative neural progenitor state [11]. In the present study, we applied this direct neuron conversion technology to the field of SMA with the aims of obtaining patient-derived motor neurons in vitro and further observing the phenotype of SMA in the induced neurons.

## RESULTS

### Induced motor neurons converted from fibroblasts

**Figure 1:**
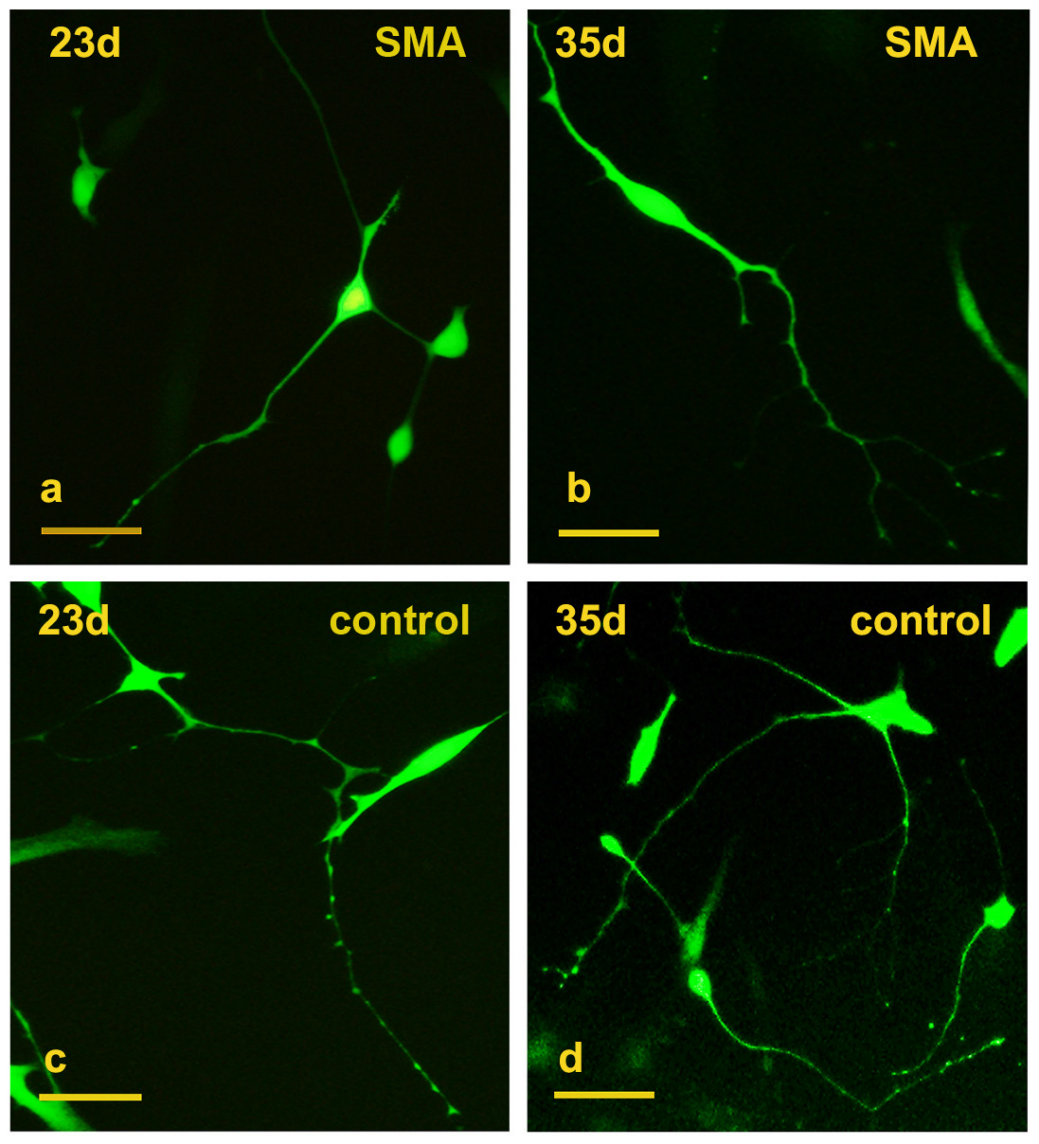
Conversion of SMA and control fibroblasts to motor neurons with 8 transcription factors **a.-b.** Typical GFP+ neuron-like cells converted from SMA fibroblasts at days 23 and 35. **c.-d.** GFP+ neuron-like cells converted from control fibroblasts at days 23 and 35. Scale bars, 100 µm.

**Figure 2:**
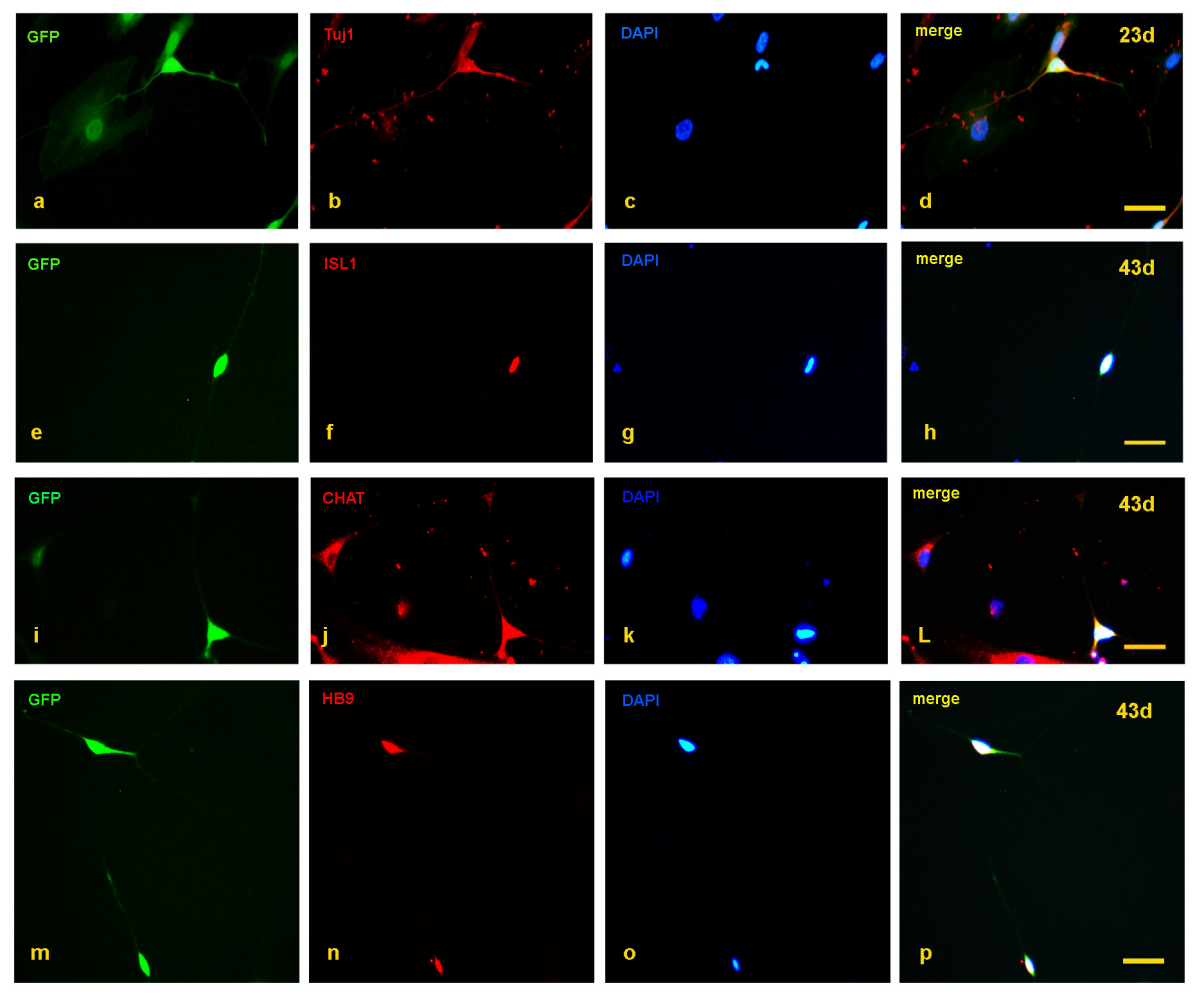
Detection of motor neuron markers with immunofluorescence staining **a.-d.** At day 23, the induced neurons expressed Tuj1. **e.-p.** At day 43, the induced neurons also expressed ISL1, CHAT, and HB9. Scale bars, 50 µm

The SMA and control fibroblasts were simultaneously infected with a cocktail of 8 lentiviruses encoding ASCL1, ISL1, NEUROD1, BRN2, HB9, LHX3, MYT1L, and NGN2. In the early induction stage (~20 days), the infected fibroblasts began to grow in a dipolar manner, and the nuclei gradually enlarged. At day 23, typical GFP positive neuron-like cells were generated (Figure 1a, 1c) and also expressed the neuronal marker Tuj1 (Figure 2a-2d). The numbers of induced neurons increased with time and at day 35, neurons with elongated and multiple neurites were observed (Figure 1b, 1d). At day 43, some GFP-positive neurons also expressed the following motor neuron markers: ISL1, CHAT and HB9 (Figure 2e-2p). In parallel groups that did not undergo transcription factor infection, neurons were not observed (Supplementary Figure 1). The induced neurons were also not stained with nestin and oligo2 (Supplementary Figure 2). Additionally, the induced motor neurons were vigorous in vitro; neuronal migration, neurite outgrowth, and the formation of neuronal connections were observed (Supplementary Figure 3). These data indicated that both the SMA and control fibroblasts could be effectively converted to motor neurons in vitro.

### Similar motor neuron conversion efficiency between the SMA and control cells

**Figure 3:**
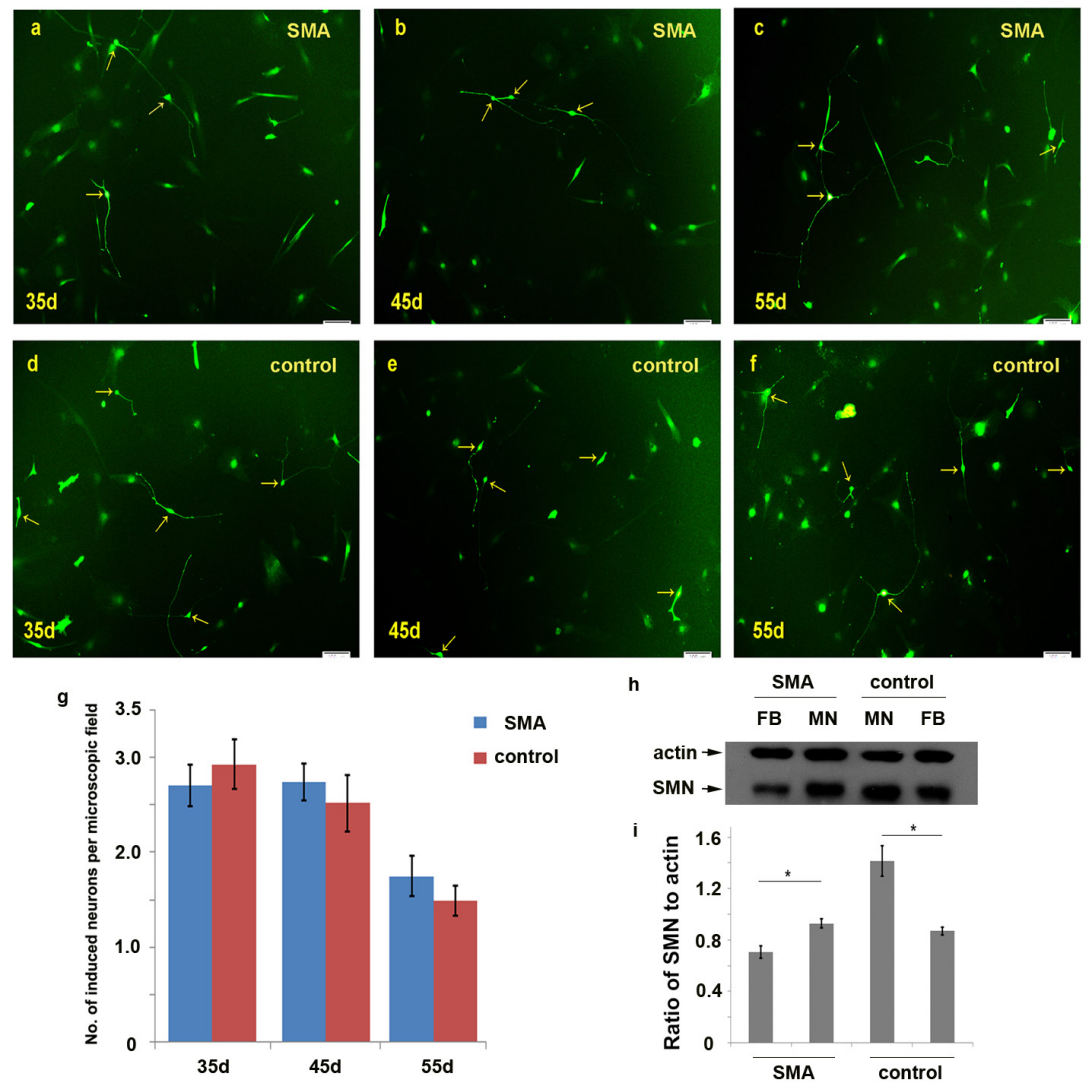
Comparison of the numbers of induced motor neurons in 10× microscopic fields between the SMA and control group **a.-f.** The induced neurons observed per microscopic field on days 35, 45 and 55 in the SMA and control groups. **g.** The average numbers of induced neurons in the SMA and control groups. The data are expressed as the means ± the SEMs. The arrow indicates an induced neuron. **h.-i.** The SMN protein levels were significantly increased in motor neurons compared with fibroblasts detected by Western blot on day 50. SMN protein levels to actin were expressed as the mean±SEM: SMA-FB, 0.71±0.05; SMA-MN, 0.93±0.03; control-MN: 1.41±0.12; control-FB: 0.87±0.03. FB: fibroblast. MN: motor neuron. * P < 0.05. Scale bars, 100 µm

We dynamically counted the numbers of induced neurons in 10× microscopic fields on days 35, 45 and 55. At least 25 different microscopic fields were randomly selected for each time point (Figure 3a-3f). The average numbers of neurons per field in SMA group were 2.70±0.22, 2.74±0.19 and 1.75±0.22 on days 35, 45 and 55, respectively. The numbers of neurons per field in the control group were 2.93±0.26, 2.52±0.30 and 1.48±0.16 on the same respective days (Figure 3g; Supplementary Table 1). We further calculated the motor neuron conversion efficiency based on the ratio of induced neurons to initially planted fibroblasts (S1 formula). The motor neuron conversion efficiencies were approximately 5.8%±1.3% and 5.5%±1.7% in the SMA and control group, respectively, and this difference was not significant. We also compared the expression levels of SMN protein between fibroblasts and induced neurons, which showed that SMN protein levels were significantly increased in motor neurons (Figure 3h, 3i).

### The SMA motor neurons exhibited a reduced neurite growth rate

**Figure 4:**
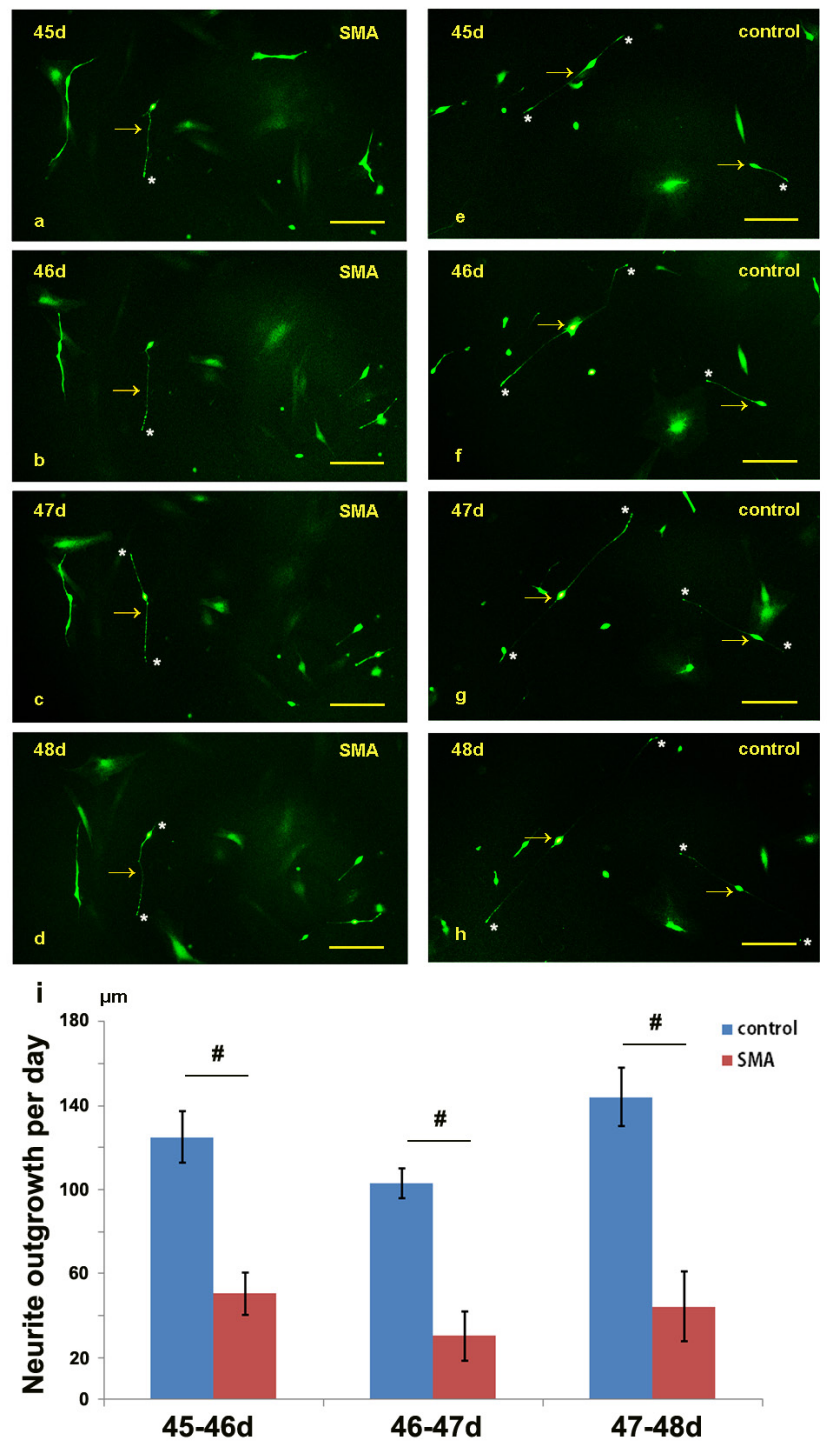
Comparison of the neurite lengths between the SMA and control group from day 45 to 48 **a.-h.** The neurite outgrowths of the SMA and control neurons were dynamically observed under a fluorescence microscope. **i.** Statistical analysis of the neurite growth rates of the SMA and control groups. The data are expressed as the means ± the SEMs. The arrow indicates an induced neuron. * indicates the terminal of a neurite. # P < 0.05. Scale bars, 100 µm

We dynamically compared the lengths of the neurites over 24 hours between the SMA and control groups from day 45 to 48 (Figure 4a-4h). In the SMA group, the average neurite outgrowth lengths were 50.53±10.09 μm, 30.32±11.73 μm, and 44.22±16.52 μm over 24 hours on days 46, 47, and 48, respectively, and the corresponding lengths in the control group were 125.02±12.10 μm, 103.04±7.03 μm, and 143.94±13.98 μm. The SMA neurons exhibited a significantly reduced neurite growth rate compared with the control groups (Figure 4i; Supplementary Table 2).

### The SMA motor neurons exhibited obvious degeneration after day 60

**Figure 5:**
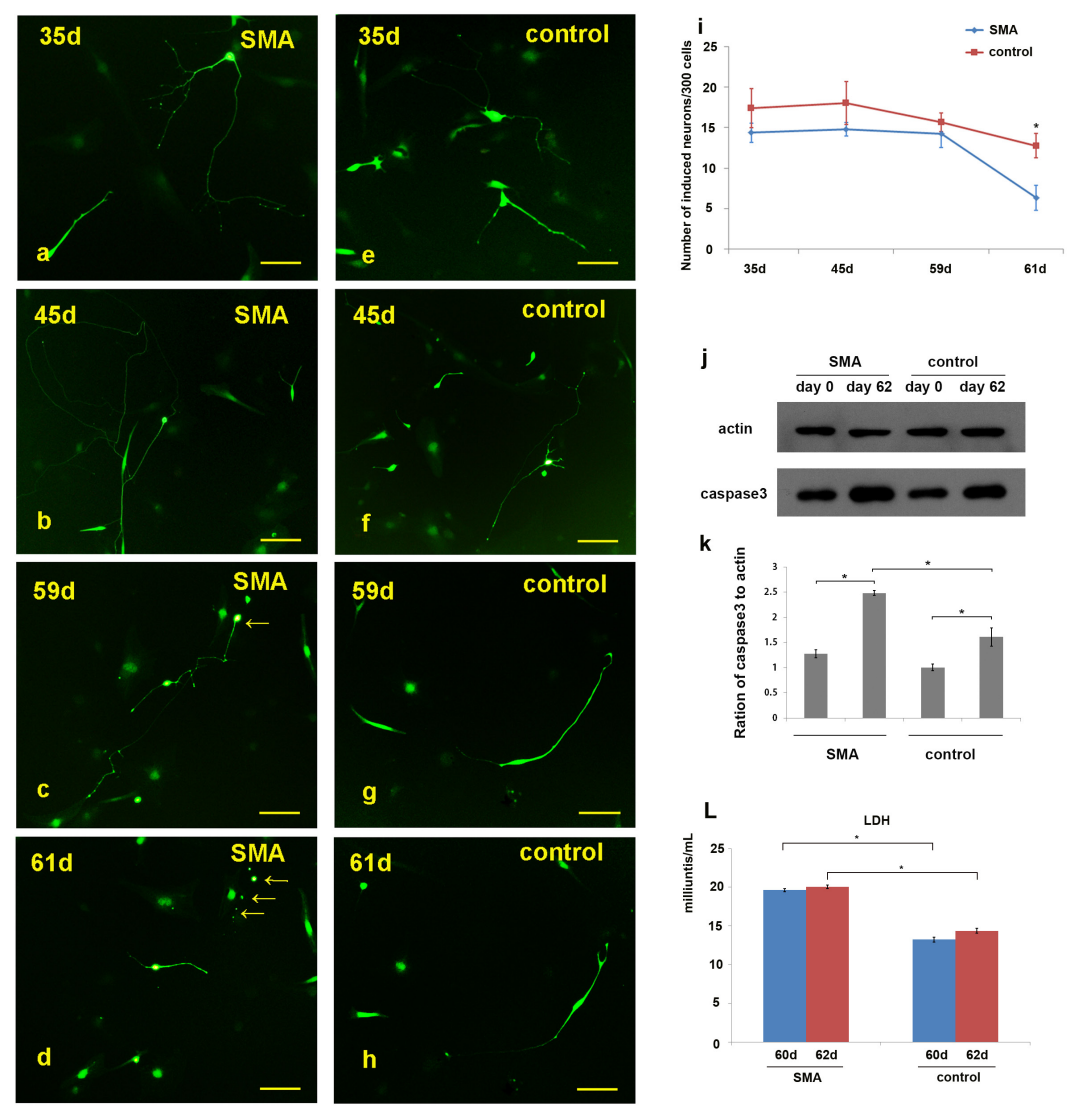
Observation of neuronal degeneration under a fluorescence microscope **a.-h.** Morphological changes in the neurites of the SMA and control neurons were dynamically observed. “**c.-d.**” and “**g.-h.**” show the same neurons in the microscopic fields. **i.** Statistical analysis of the induced neurons per 300 cells revealed the following results: 14.4±1.2 (SMA-35 d), 14.8±0.8 (SMA-45 d), 14.2±1.6 (SMA-59 d), 6.3±1.5 (SMA-61 d), 17.4±2.4 (control-35 d), 18.0±2.6 (control-45 d), 15.7±1.2 (control-59 d), and 12.8±1.5 (control-61 d). **j.-k.** The caspase 3 levels in SMA group compared with control group on day 62. Caspase3 protein levels to actin were expressed as the mean±SEM: SMA-day 0, 1.27±0.08; SMA-day 62, 2.48±0.05; control-day 0: 1.00±0.06; control-day 62: 1.61±0.18. **l.** The lactate dehydrogenase (LDH) activity detected between SMA and control group at day 60 and day 62. LDH activity was expressed as the mean±SEM: SMA-day 60, 19.63±0.19; SMA-day 62, 20.04±0.22; control-day 60: 13.23±0.33; control-day 62: 14.37±0.31. * P < 0.05. Scale bars, 100 µm

After day 60, obvious disintegration of the neurites was observed in the SMA motor neurons in vitro (Figure 5a-5h; Supplementary Figure 4 ). We also calculated the numbers of neurons in per 300 cells on days 35, 45, 59 and 61 (300 cells/group, ≥3 groups). In the SMA group, the average numbers of neurons per 300 cells were 14.4±1.2, 14.8±0.8, 14.2±1.6 and 6.3±1.5 on days 35, 45, 59 and 61, respectively. In the control group, the corresponding numbers of neurons were 17.4±2.4, 18.0±2.6, 15.7±1.2 and 12.8±1.5 (Figure 5i; Supplementary Table 3). There were no significant differences between the SMA and control groups on days 35, 45 and 59, but the number of neurons was significantly reduced on day 61 in the SMA group (t = 6.612, p = 0.000). The caspase 3 levels were significantly increased in SMA group compared with control group at day 62 (Figure 5j-5k), and the SMA group also showed a high lactate dehydrogenase (LDH) activity detected in the medium at day 60 and day 62 serially (Figure 5l). We further employed a small molecular compound, SAHA, which is a kind of HDACi could increase the expression of SMN protein. SAHA was treated at day 62, with a final concentration of 1.0 μM. We observed the morphological changes every 12 hours; which turned out that SAHA could maintain the growth of induced neurons (Supplementary Figure 5). Compared with the number of neurons at day 61, no significant reduce was observed at day 65 (Supplementary Figure 6).

## DISCUSSION

SMA is pathologically characterized by the selective degeneration and loss of motor neurons in the spinal cord. Traditionally, it was impossible to obtain patient-originated and SMA-related motor neuron samples. The spring cellular conversion technology made it possible to obtain induced motor neurons from SMA patients in vitro. In this study, we established a feeder-free conversion procedure to obtain SMA patient-specific induced motor neurons directly from fibroblasts. These induced motor neurons exhibited typical neural morphologies and expressed the hallmarks of TUJ1, HB9, ISL1 and CHAT. Additionally, these neurons were vigorous in culture and possessed the capabilities of neuronal migration, neurite outgrowth, and formation of neuronal connections.

The advantage of cellular trans-differentiation lies in that we could generate patient-derived motor neurons directly with simple and time-saving procedure. Although the fibroblasts could be converted to motor neurons in vitro, the conversion efficiency was still low. Recently, increasing attention has been focused on the development of high-efficiency conversion systems. Li X and colleagues found that mouse fibroblasts can be efficiently converted into functional neurons with small molecules, such as forskolin, ISX9, CHIR99021, and I-BET151, and named these neurons chemical-induced neurons (CiNs) [12]. Hu W and colleagues also generated CiNs from normal and Alzheimer’s disease human fibroblasts via a chemical cocktail of valproic acid, CHIR99021, RepSox, forskolin, SP600125, GO6983, and Y-27632 [13]. This transgene-free and chemical-only approach offers a new avenue for the generation of induced neurons in vitro. Liu ML et al. also reported the rapid and efficient conversion of ALS patient fibroblasts to motor neurons via the overexpression of NGN2, SOX11, ISL1 and LHX3 with the addition of the extrinsic factors forskolin and dorsomorphin in neuron-induction media [14]. In the present study, the motor neuron conversion efficiencies were approximately 5.8% and 5.5% in the SMA and control groups, respectively, which could likely be increased by the addition of some specific chemical molecules in the future.

SMA is pathologically characterized by the degeneration of motor neurons; however, the underlying molecular mechanism responsible for the motor neuron vulnerability remains unknown. Chang T et al. observed that the iPSCs-derived motor neurons of SMA patients showed delayed neurite outgrowth that can be restored by the ectopic expression of SMN [15]. Corti S et al. reported that the axon lengths of SMA-iPSC-derived motor neurons were shorter than those of a wild-type group, and the growth cones were also smaller in the SMA group [16]. Sareen D et al. also reported that SMA-iPSC-derived motor neurons exhibit increased Fas ligand-mediated apoptosis and caspase activation [17]. Similarly, in the present study, we also dynamically calculated the lengths of the neurites from day 45 to 48, and the results indicated that the SMA induced neurons exhibited a significant decrease in neurite outgrowth per day compared with the control group. Additionally, the SMA induced neurons exhibited neural degeneration after day 60, and the number of motor neurons was significantly decreased compared with control group. In induced motor neurons, we observed the difference in the aspect of neurite outgrowth and neural degeneration; they represented part of SMA phenotype, and it shed some light on the molecular mechanism of SMA. From this study, the SMA induced motor neurons seemed to reduce neurite growth and increase neuron degeneration in vitro.

In summary, we established a feeder-free direct conversion procedure to obtain SMA patient-specific induced motor neurons from fibroblasts. These SMA patient-derived induced neurons reproduced some SMA-specific features in vitro and thus could be used as a cell model in pathogenesis research and drug screening.

## MATERIALS AND METHODS

### Subjects

Fibroblast cell lines were obtained from type III SMA patients and non-motor neuron disease controls. All the SMA patients exhibited extensive neurogenic lesions on electromyography (EMG) tests. The homozygous deletions exon 7 and exon 8 in SMN1 were tested with polymerase chain reaction-restricted fragment length polymorphism (PCR-RFLP). Written informed consent was obtained from each participant. This study was approved by the Ethics Committee of First Affiliated Hospital of Fujian Medical University.

### Plasmid cloning and lentivirus production

The cDNA sequences of ASCL1, ISL1, NEUROD1, BRN2, HB9, LHX3, MYT1L and NGN2 were transferred into lentiviral tet-controlled vectors (pLV-EF1α-eGFP-TRE-cDNA; purchased from SiDanSai Biotechnology Co., Ltd., Shanghai, China). The lentiviral plasmids were then transduced into 293T cells with the aid of helper plasmids (Δ8.91 and pVSVG) and Fugene (Roche). The lentivirus supernatants were collected at 48 and 72 hours after transduction. The viral titers were tested and then used in the subsequent experiments.

### Direct motor neuron induction

The fibroblasts were expanded for 2 or 3 passages before being used in the experiments. The cells were infected with lentiviruses encoding ASCL1, ISL1, NEUROD1, BRN2, HB9, LHX3, MYT1L and NGN2 in addition to rtTA and polybrene in 6-well plates coated with poly-L-ornithine and laminin (both from Sigma), and the date was recorded as day 0. On day 1, the lentiviruses were removed, and the infected fibroblasts were kept in F10 medium supplemented with FBS (20%) and doxycycline (DOX, 2 μg/ml) for 2 days. On day 3, the medium was switched to N3 medium containing the following: DMEM/F12 supplemented with ITS-G (1×), progesterone (20 nM), putrescine hydrochloride (100 nM), FGF2 (10 ng/ml), P/S (1×), and DOX (2 μg/ml). The medium was changed every 2 days. On day 20, when neurons were found to exhibit neuronal morphology, the induction medium was switched to maturation medium, i.e., Neurobasal-A (Gibco) supplemented with B27 (1×), AA (200 ng/ml), FGF2 (10 ng/ml), BDNF (10 ng/ml, Gibco), GDNF (10 ng/ml, Gibco), IGF1 (10 ng/ml, Gibco), and cAMP (1 µM, Sigma). The medium was changed every 2 days.

### Immunofluorescence staining

The cells were fixed in 4% paraformaldehyde for 10 min at room temperature and then blocked in PBS supplemented with 1% BSA, 4% normal serum and 0.4% Triton-X-100 at 4oC overnight. After blocking, the cells were incubated with primary antibodies at 4oC overnight. After washing with PBS, the cells were incubated and fluorescence-labeled with secondary antibody and counterstained with DAPI. Finally, the cells were visualized using an inverted fluorescence microscope (Olympus, IX71). The primary antibodies adopted in this study included β3 Tubulin (Tuj-1, Santa-Cruz), HB9 (Santa-Cruz), CHAT (Proteintech) and ISL1 (DSHB).

### Induced motor neuron counts and neurite length assay

Motor neurons were observed under a fluorescence microscope at different time points. The numbers of motor neurons were counted based on GFP-positive, typical neural morphologies and neuron marker expression under 10× microscopic field. We then counted the average numbers of neurons per field in SMA group and control group on days 35, 45 and 55, respectively. The motor neuron conversion efficiency was calculated based on the ratio of induced neurons to initially planted fibroblasts on day 35, 45 and 55, and finally compared the motor neuron conversion efficiency based on these three time points. To compare the neurite outgrowth rates between the SMA and control cells, we dynamically observed the lengths of the neurites from day 45 to 48. The lengths of the neurites were calculated according to the scale bars. For neurons had multiple neurite processes, we compared the lengths of all neurites.

The total proteins were extracted at different time point. The SMN protein and caspase 3 were detected by Western blot. The lactate dehydrogenase (LDH) activity was detected in the medium at day 60 and day 62 by LDH Activity Kit (Sigma-Aldrich, Catalog #: MAK066). SMA induced motor neurons were treated with SAHA at day 62 (1.0 μM), and the morphological changes were observed every 12 hours.

### Statistical analysis

An independent-samples T test was used to analyze the difference in the numbers of induced neurons between the SMA and control samples. P < 0.05 (two-tailed) was considered statistically significant.

## SUPPLEMENTARY MATERIALS FIGURES AND TABLES


