# Supplementary material for: Modeling the phenotype of spinal muscular atrophy by the direct conversion of human fibroblasts to motor neurons
Source: Oncotarget. 2017 Jan 13;8(7):10945–53. doi: 10.18632/oncotarget.14641 (PMC5355236; doi:10.18632/oncotarget.14641)
Supplement: Supplementary file 1 [file oncotarget-08-10945-s001.pdf]

Modeling the phenotype of spinal muscular atrophy by the direct conversion of human fibroblasts to motor neurons

Qi-Jie Zhang[1,*], Jin-Jing Li[1,*], Xiang Lin[1], Ying-Qian Lu[1], Xin-Xin Guo[1], En-Lin Dong[1], Miao Zhao[1], Jin He[1], Ning Wang[1,2] and Wan-Jin Chen[1,2]

[1] Department of Neurology and Institute of Neurology, First Affiliated Hospital, Fujian Medical University, Fuzhou, China

[2] Fujian Key Laboratory of Molecular Neurology, Fuzhou, China

[*] These authors have contributed equally to this work

Correspondence to: *Wan-Jin Chen*, ***email***: *wanjinchen75@fjmu.edu.cn*

Keywords: direct reprogramming; fibroblast; induced motor neuron; spinal muscular atrophy

Received: *June 08, 2016* ***Accepted***: *November 22, 2016* ***Published***: *January 13, 2017*

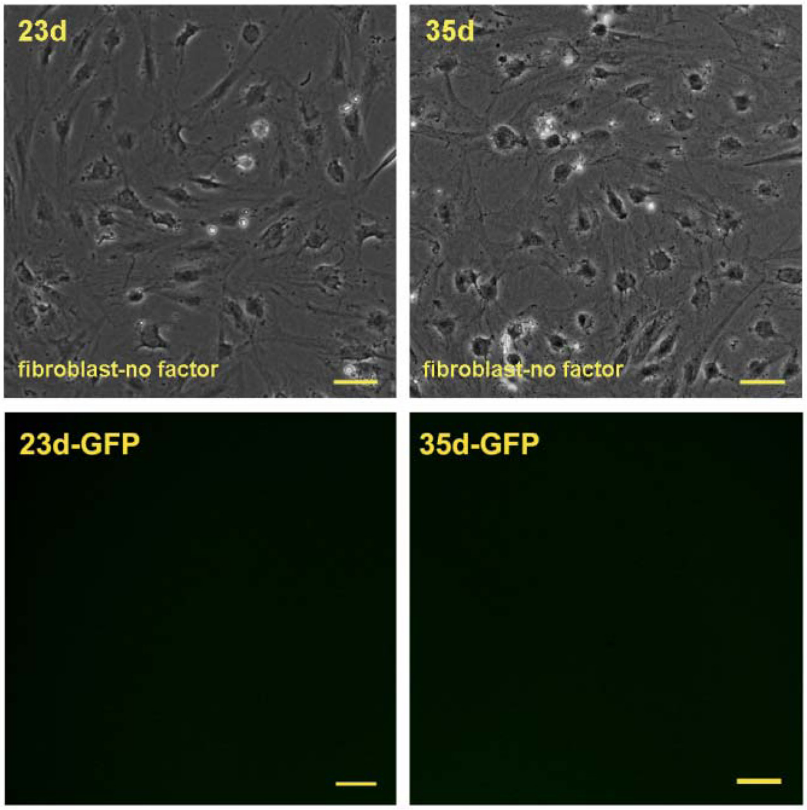

Supplementary Figure 1: Neuron-like cells were not observed in the fibroblasts without transcription factor infection at day 23 and 35. Scale bars, 100μm.

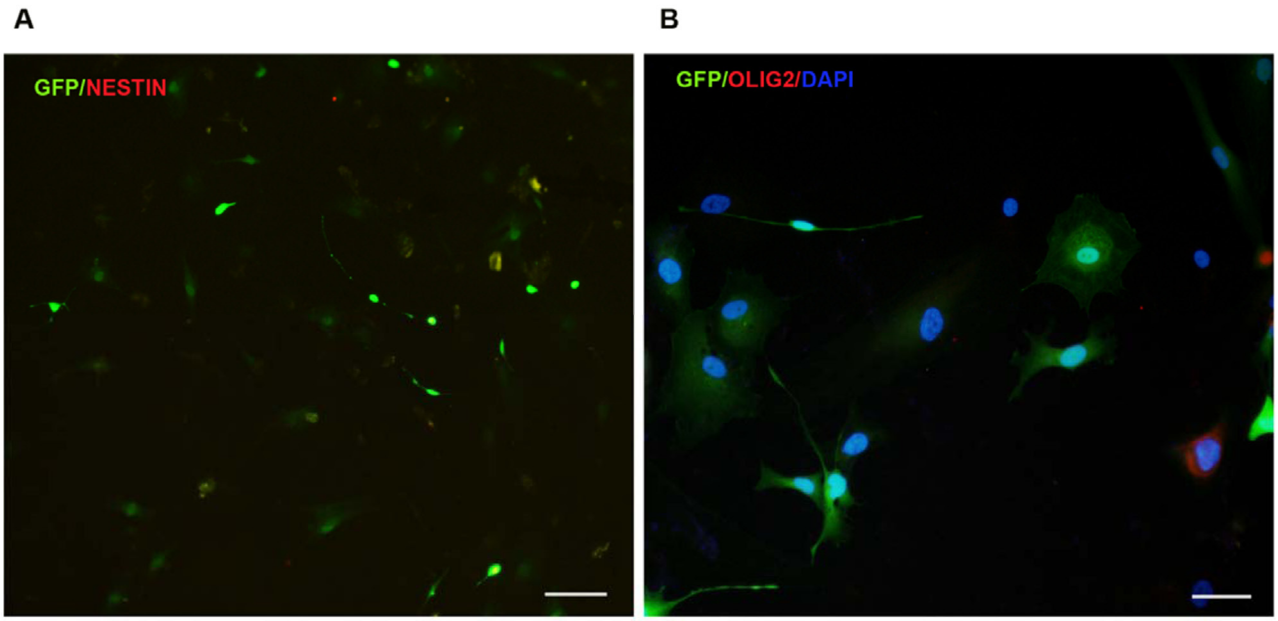

Supplementary Figure 2: The induced neurons were not expressed nestin and olig 2 at day 45. Scale bars: a, 100μm, b, 50μm.

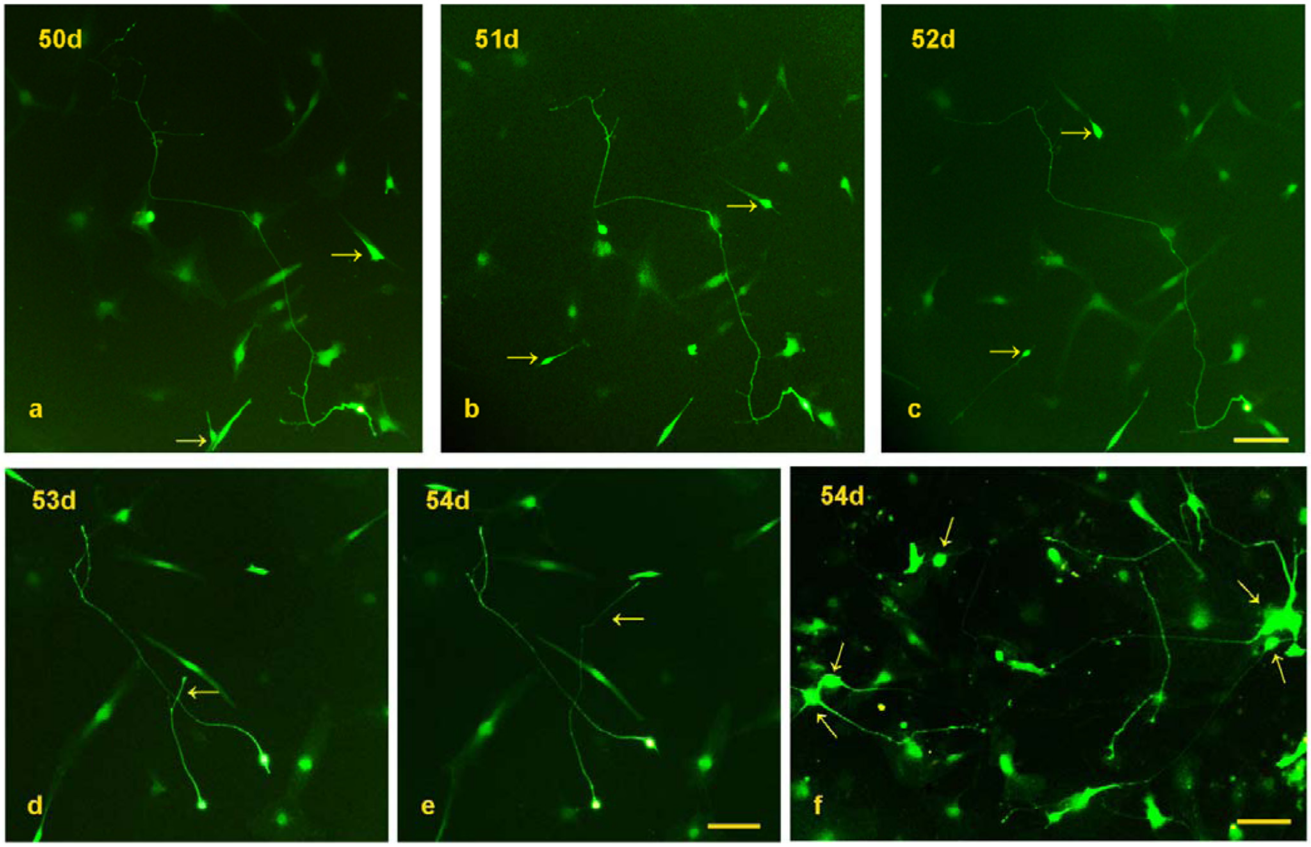

Supplementary Figure 3: The induced neurons were vigorous in culture. (a-c) neuron migration. (d-e) outgrowth of neurite. (f) formation of neuron net. Arrow indicates the induced neuron. Scale bars, 100μm.

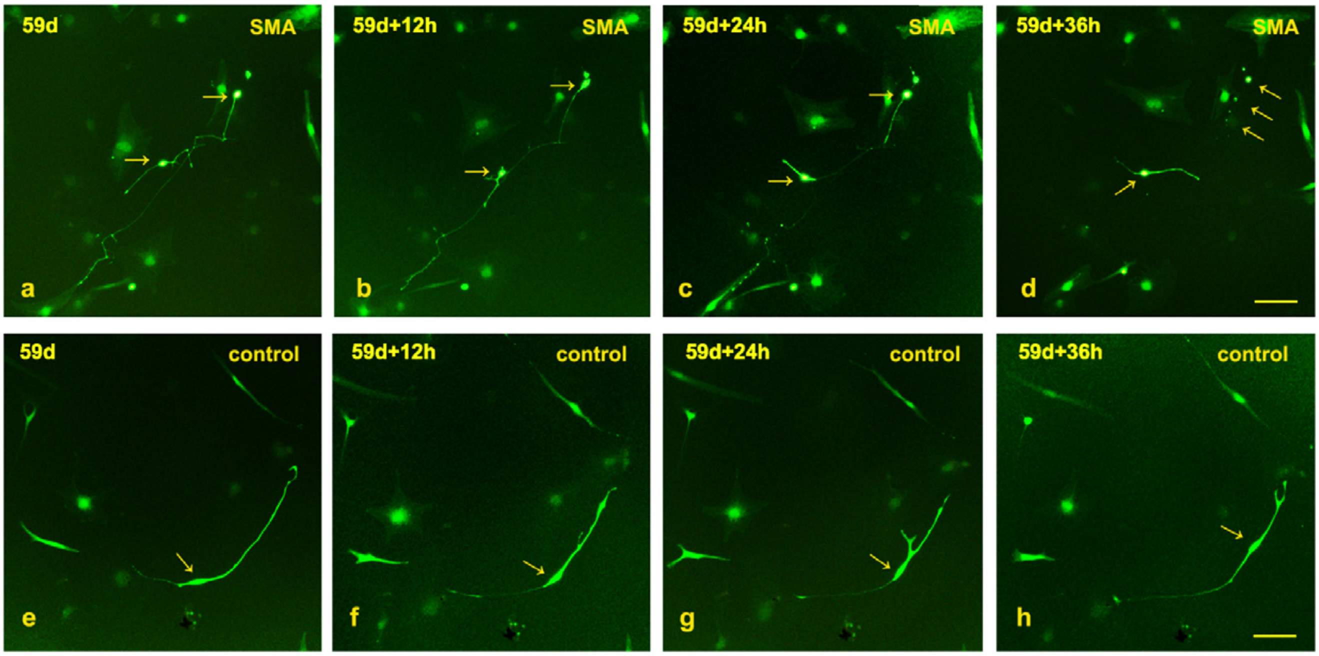

Supplementary Figure 4: Dynamical observation of neuronal morphologies of induced neurons under fluorescence microscope from day 59 to 61. (a-d) SMA-induced neurons. (e-h) control-induced neurons. Scale bars: 100μm.

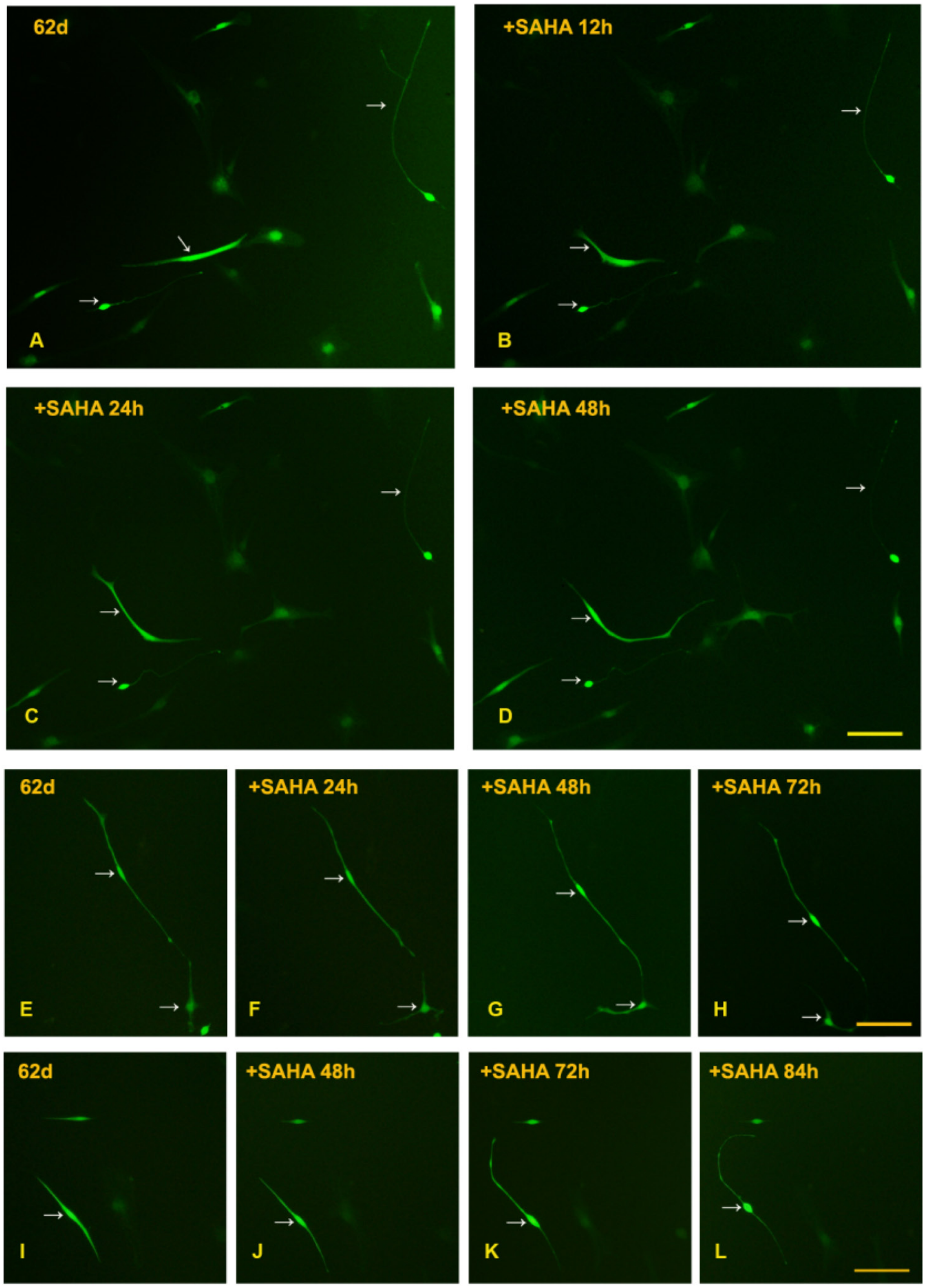

Supplementary Figure 5: SAHA treatment could maintain the growth of induced SMA motor neurons. SAHA was treated at day 62, and the cellular morphological changes were observed every 12 hours. Scale bars: 100μm.

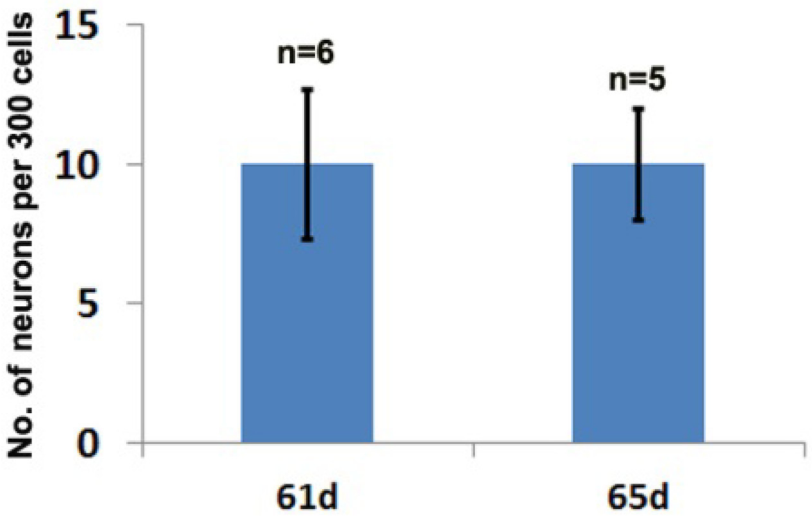

Supplementary Figure 6: Comparison of motor neurons at day 61and day 65 in SMA group. The number of neurons per 300 cells were 10±2.7 and 10±2.0 respectively.

S1 Table. The number of induced neurons under 10× microscopic field at day 35, 45 and 55

Group	time	Number of induced neurons per microscopic field																																								mean	sem
SMA	35d	4	3	3	1	4	0	4	3	3	2	3	4	2	4	4	4	1	5	4	1	4	3	1	2	1	4	3	3	4	3	3	2	1	4	1	1	1	/	/	/	/	2.7	0.22
	45d	3	3	3	4	3	2	2	1	2	3	2	4	4	4	2	3	4	1	3	2	3	5	2	1	2	4	2	4	1	3	3	/	/	/	/	/	/	/	/	/	/	2.7	0.19
	55d	3	2	0	1	1	3	3	2	0	2	2	1	2	4	2	3	0	3	0	1	3	0	1	2	2	2	1	3	/	/	/	/	/	/	/	/	/	/	/	/	/	1.8	0.22
control	35d	2	2	0	1	2	5	0	2	3	1	4	2	3	3	6	4	3	3	3	3	3	5	2	5	1	2	4	5	1	2	1	6	1	4	3	3	7	4	5	1	3	2.9	0.26
	45d	5	4	5	1	1	4	1	2	1	2	2	1	2	4	1	4	3	3	1	2	0	3	6	2	4	3	1	/	/	/	/	/	/	/	/	/	/	/	/	/	/	2.5	0.30
	55d	2	2	1	1	1	1	0	2	3	2	1	1	1	1	3	2	2	0	3	0	1	1	2	2	3	2	0	1	1	2	1	3	1	/	/	/	/	/	/	/	/	1.5	0.16

S1 Formula. The conversion rate of motor neuron

$$\text{Conversion rate} = \frac{\text{Number of MNs} \times \dfrac{\text{Area of 6-well plate}}{\text{Area of microscopic field}}}{\text{Number of fibroblasts}}$$

Area of 6-well plate = 9.6 cm^2

Area of each microscopic field = 0.013 cm^2

Number of fibroblasts each well = 3×10^4

5

S2 Table. The neurite outgrowth per 24 hours from day 45 to 48

group	day	neurite outgrowth per neuron (μm)						mean	sem
SMA	45-46	15.2	60.6	90.9	45.5	45.5	45.5	50.5	10.09
	46-47	7.6	7.6	37.9	15.2	30.3	83.3	30.3	11.73
	47-48	15.2	37.9	7.6	15.2	98.5	90.9	44.2	16.52
control	45-46	90.9	159.1	106.1	140.2	128.8	/	125.0	12.10
	46-47	106.1	128.8	90.9	98.5	90.9	/	103.0	7.03
	47-48	121.2	136.4	197	143.9	121.2	/	143.9	13.98

S3 Table. The number of induced neurons per 300 cells at day 35, 45, 59 and 61

group	day	number of induced neurons per 300 cells							
SMA	35	14	14	13	16	15	16	13	14
	45	14	15	16	14	15	/	/	/
	59	14	13	14	13	17	/	/	/
	61	7	6	9	5	6	5	/	/
control	35	15	20	16	16	20	/	/	/
	45	19	20	15	/	/	/	/	/
	59	15	15	17	/	/	/	/	/
	61	15	12	12	12	/	/	/	/
